# Analysis of the Radiosensitivity Index in Paired Preneoadjuvant and Postneoadjuvant Therapy Triple-Negative Breast Cancer

**DOI:** 10.1016/j.ijrobp.2025.09.038

**Published:** 2025-09-25

**Authors:** Shane R. Stecklein, Roberto Salgado, Julia R. White, Bruce F. Kimler, Rachel Yoder, Joshua M. Staley, Anne P. O’Dea, Lauren E. Nye, Deepti Satelli, Gregory J. Crane, Rashna Madan, Maura F. O’Neil, Andrew K. Godwin, Harsh Pathak, Qamar J. Khan, Joyce O’Shaughnessy, Priyanka Sharma

**Affiliations:** a Department of Radiation Oncology, University of Kansas Medical Center, Kansas City, Kansas;; b Department of Pathology and Laboratory Medicine, University of Kansas Medical Center, Kansas City, Kansas;; c Department of Cancer Biology, University of Kansas Medical Center, Kansas City, Kansas;; d The University of Kansas Cancer Center, Kansas City, Kansas;; e Department of Breast Radiation Oncology, University of Texas MD Anderson Cancer Center, Houston, Texas;; f Department of Pathology, ZAS Hospital, Antwerp, Belgium;; g Division of Research, Peter MacCallum Cancer Center, Melbourne, Victoria, Australia;; h Division of Medical Oncology, Department of Internal Medicine, University of Kansas Medical Center, Kansas City, Kansas;; i Kansas Institute for Precision Medicine, University of Kansas Medical Center, Kansas City, Kansas;; j Texas Oncology/Baylor Charles A. Sammons Cancer Center, Dallas, Texas

## Abstract

**Purpose:**

The radiosensitivity index (RSI) is a validated gene expression-based biomarker that can predict intrinsic radiosensitivity and has been shown to be associated with local control in patients with triple-negative breast cancer (TNBC) treated with upfront surgery. Currently, most patients with TNBC receive neoadjuvant systemic therapy (NAST). Whether the RSI predicts response to NAST and how the RSI and predicted radiosensitivity are altered by exposure to NAST is unknown.

**Methods and Materials:**

Total RNA was extracted from pretreatment core needle biopsy specimens from 197 patients with TNBC treated on the NeoSTOP (NCT02413320) and NeoPACT (NCT03639948) trials. Total RNA was also extracted from paired post-NAST (residual disease) tumor tissue in 58 patients. A published algorithm using 10 genes was used to compute the RSI for each tumor. Stromal tumor-infiltrating lymphocytes (sTILs) were scored on pretreatment and post-NAST samples by an expert breast pathologist according to international consensus guidelines. CIBERSORTx was used to impute leukocyte fractions in samples using RNA-sequencing data.

**Results:**

Cluster analysis of RSI genes in pretreatment samples revealed immune-depleted and immune-enriched groups, and this classification was strongly associated with sTIL infiltration (*P* < .0001) and likelihood of achieving pathologic complete response (pCR) (*P* = .001). RSI showed associations (false discovery rate *q* < 0.01) with M0 and M1 macrophages, CD4+ memory resting, CD4+ memory activated, CD8+, and follicular helper T-cells, activated natural killer cells, naïve and memory B cells, and resting dendritic cells on CIBERSORTx leukocyte deconvolution. In the entire cohort, NAST-induced change in RSI was variable, but among initially RSI-immune-enriched tumors that did not achieve pCR, there was a significant decrease in predicted radiosensitivity between paired pretreatment and post-NAST samples. NAST-induced reduction in sTILs and naïve B cells may be associated with this decrease in radiosensitivity.

**Conclusions:**

Pretreatment RSI cluster identity is associated with the degree of immune enrichment and response to NAST in TNBC. Initially immune-enriched TNBCs that do not achieve a pCR to NAST exhibit a decrease in predicted radiosensitivity compared with paired pretreatment tumors.

## Introduction

Radiation therapy is a critical component of curative-intent therapy for patients with breast cancer who undergo breast conserving surgery and for those with involvement of regional lymph nodes. In these contexts, radiation therapy reduces the risk of locoregional recurrence, reduces the risk of distant metastasis, and improves survival.^1,2^ Although the use of moderately or ultrahypofractionated radiation therapy regimens has improved the convenience of adjuvant radiation therapy for patients with breast cancer,^3–5^ these regimens were designed to be isoeffective for tumor control compared with the historical standard of 50 Gy in 25 fractions.

Completed prospective efforts to personalize radiation therapy in breast cancer have focused on using clinicopathologic features^6–8^ and response to neoadjuvant therapy^9^ to identify patients at low risk of locoregional recurrence and/or breast cancer mortality without radiation therapy. Ongoing prospective randomized studies have incorporated genomic biomarkers to identify biologically low-risk patients who are likely to achieve negligible benefit from adjuvant radiation therapy (NCT04852887 and NCT03488693). These studies have all focused on attempting to define groups of patients who can safely omit radiation therapy. The probability of controlling a tumor at a given dose and fractionation of radiation therapy is deterministic, and depends on the number of malignant clonogens present, and the intrinsic radiosensitivity of these clonogens. As such, the dichotomous (ie, yes vs no) application of adjuvant radiation therapy likely leads to overtreatment of some patients and undertreatment of others. With validated biomarkers of radiosensitivity, it should be possible to optimize the radiation therapy dose necessary for an individual patient to maximize the probability of tumor control while mitigating unnecessary toxicity.

A systems biology approach to understanding tumor cell intrinsic radiosensitivity through in vitro modeling in cancer cell lines led to the development of the radiosensitivity index (RSI).^10^ This 10-gene expression signature has been extensively validated as a reliable predictor of radiosensitivity in a variety of human tumors, including breast cancer,^11–18^ and it has been integrated in to the linear quadratic model^19^ of radiation cell kill to generate the genomically adjusted radiation dose (GARD) paradigm for personalized radiation therapy doses in the clinical setting.^20^ Recent work in triple-negative breast cancer (TNBC) showed that GARD was associated with local control following adjuvant radiation therapy and that this could potentially be used to optimize radiation therapy doses for individual patients in this setting.^21^ The 2 cohorts of patients with TNBC in this analysis were all treated with upfront surgery and patients did not uniformly receive adjuvant chemotherapy.^22,23^ Currently, most patients with TNBC receive chemoimmunotherapy in the neoadjuvant setting,^24^ and response to neoadjuvant systemic therapy is a powerful predictor of both locoregional and distant recurrence risk.^25,26^ To date, RSI has not been examined in the context of predicting pathologic response to neoadjuvant systemic therapy, nor has it been determined if or how NAST exposure changes RSI and predicted radiosensitivity.

## Methods and Materials

### Study population

Patients with stage I-III TNBC who were treated with neoadjuvant systemic therapy on NeoSTOP (NCT02413320)^27^ or NeoPACT (NCT03639948)^28^ were included in this study. The patient demographics, treatment details, and disease-related outcomes for patients enrolled on both studies have been reported.

### Gene expression analysis

RNA was extracted from formalin-fixed paraffin-embedded pretreatment and posttreatment tumor specimens and subjected to RNA sequencing using the Illumina Tru-Seq RNA Exome Panel (Almac). Raw sequence data were converted from binary base call to FASTQ format, and demultiplexed on the Illumina BaseSpace Sequence Hub. FASTQ files were processed through the Almac RNAExome bioinformatics pipeline to generate both sequence QC metrics, such as total read counts, GC content and duplication rate, and alignment QC metrics such as mapping rate. Reads were aligned to the reference human genome (hg19), samples were normalized using fragments per kilobase million, and expression levels were log(2) transformed to generate an expression matrix. CIBERSORTx analysis was performed, as previously described.^29^

### Stromal tumor-infiltrating lymphocyte assessment

Stromal tumor-infiltrating lymphocytes (sTILs) were assessed on pretreatment core needle biopsy specimens and residual disease surgical specimens by a single expert breast pathologist (R.S.) according to international consensus standards.^30^

### RSI and GARD calculation

The RSI was generated as previously described. Briefly, each of the 10 genes were ranked from highest (10) to lowest (1) and RSI was calculated according to the published rank-based algorithm,^10,16^ where RSI = −0.0098009 * AR + 0.0128283 * JUN + 0.0254552 * STAT1 – 0.0017589 * PRKCB – 0.0038171 * RelA + 0.1070213 * ABL1 – 0.0002509 * SUMO1 – 0.0092431 * PAK2 – 0.0204469 * HDAC1 – 0.0441683 * IRF1. Because a negative *RSI* would lead to an undefined *α*, and thus prevent calculation of GARD,^20^ RSI was normalized to the full possible range of RSI (−0.6050984 to +1.219103) and scaled such that RSI was always between 0 and 1 for all samples according to RSI_scaled_ = (RSI – RSI_min_)/(RSI_max_ – RSI_min_). Using the linear quadratic formalism (Surviving Fraction (SF) = *e*^−*nd*(*α*+*βd*)^), a patient-specific *α* was derived by substituting the patient-specific RSI_scaled_ for SF with the assumption that dose (*d*) was 2.0 Gy, there was a single fraction (*n* = 1), and *β* was constant at 0.05 Gy^−1^. The patient-specific *α* is thus defined as *α* = −0.5 ln(RSI_scaled_) −0.1 and the RSI_scaled_ is equivalent to the predicted tumor cell survival after an acute exposure of 2 Gy (SF_2Gy_).

### Single-cell analysis

Single-cell RSI gene analysis was examined in published single-cell RNA-sequencing data^31^ using the Broad Institute’s Single Cell Portal (https://singlecell.broadinstitute.org/single_cell).

### Statistical analysis and data presentation

For hierarchical clustering, gene expression scores for each gene were Z-scored among all samples and clustering was performed using Manhattan distance with Ward linkage. Silhouette analysis^32^ was used to identify the optimal number of groups for k-means clustering. Comparisons between groups were conducted using parametric or nonparametric tests, with pairing if appropriate, and details for each analysis are provided in the figure legends. Statistical analyses were performed in *R* or SPSS. Graphs and plots were generated in *R* or GraphPad Prism and final formatting was performed in Adobe Illustrator.

## Results

### Patient characteristics

Demographic, tumor, and treatment characteristics from the 197 evaluable patients in NeoSTOP and NeoPACT are shown in Table 1. Given the nearly identical eligibility criteria for these 2 sequential trials, trial-specific systemic therapy was the only significant difference between the 2 study populations.

### Cluster analysis by RSI genes identifies immune-enriched and immune-depleted TNBCs and implicates degree of immune enrichment as a critical determinant of RSI score

**Fig. 1.**
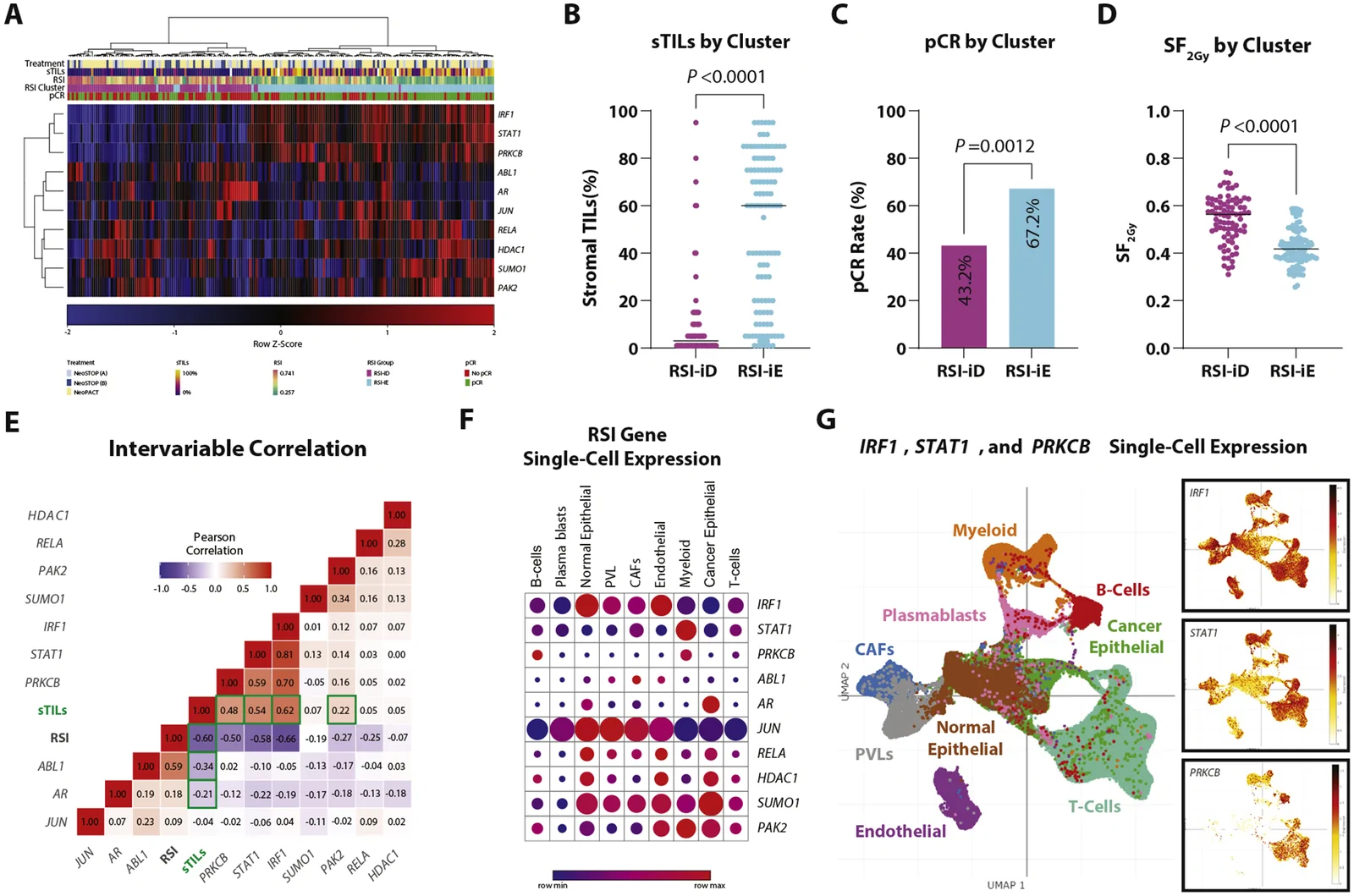
(A) Unsupervised hierarchical clustering of 197 pretreatment TNBC samples by 10 RSI genes. (B) sTILs by RSI-iD and RSI-iE groups; *P* is Mann-Whitney *U*test. (C) pCR rate in RSI-iD and RSI-iE clusters; *P* is Fisher’s exact test. (D) Predicted SF_2Gy_ by RSI-iD and RSI-iE groups; *P* is Mann-Whitney *U* test. (E) Pearson correlation coefficients among RSI genes, computed RSI, and sTILs. Cells with green outline denote significant (Benjamini-Hochberg [BH] *q* < 0.01) associations with sTILs. (F) Single-cell expression of 10 RSI genes in defined cellular components of human breast cancers. The size of the circle reflects the percentage of cells expressing the gene, and the color reflects the scaled mean expression within that cell population. (G) The large panel shows a Uniform Manifold Approximation and Projection (UMAP) plot of single cells composing human breast cancers. The small plots show expression of *IRF1, STAT1*, and *PRKCB* within these various cellular compartments. *Abbreviations:* CAF = cancer-associated fibroblast; pCR = pathologic complete response; PVL = perivascular-like cell; RSI = radiosensitivity index; RSI-iD = RSI immune-depleted; RSI-iE = RSI immune-enriched; sTIL = stromal tumor-infiltrating lymphocyte; TNBC = triple-negative breast cancer.

Unsupervised hierarchical clustering of 197 pretreatment TNBC samples by the 10 RSI genes demonstrated clear segregation into 2 clusters driven by coordinated expression of *IRF1, STAT1*, and *PRKCB* (Fig. 1A). Silhouette analysis confirmed that expression of the 10 RSI genes was best characterized by 2 clusters, so k-means clustering was performed to generate dichotomous classifications for all 197 samples. The 2 clusters exhibited markedly different degrees of sTIL infiltration (median of 3% vs 60%; *P* < .0001; Fig. 1B), and we thus termed these 2 clusters RSI immune-depleted (RSI-iD) and RSI immune-enriched (RSI-iE). The rate of pathologic complete response (pCR) was significantly higher in RSI-iE compared with RSI-iD tumors (67.2% vs 43.2%, *P* = .001; Fig. 1C), consistent with prior reports showing that various measures of immune enrichment predict pCR.^28,33^ RSI-iE cluster identity remained an independent predictor of pCR (OR, 2.71; 95% CI, 1.51–4.88; *P* = .001) in a binary logistic regression accounting for differences in neoadjuvant systemic therapy. We also observed that pretreatment RSI-iE tumors had significantly higher predicted radiosensitivity (ie, lower tumor cell survival after an acute exposure of 2 Gy (SF_2Gy_)) compared with RSI-iD tumors (median SF_2Gy_ of 0.42 vs 0.56; *P* < .0001), though there was still a large range of predicted SF_2Gy_ within RSI clusters (Fig. 1D). sTILs were significantly positively correlated with *IRF1, STAT1, PRKCB*, and *PAK2* expression and significantly inversely correlated with *ABL1* and *AR* expression, and with the RSI (Fig. 1E). Although the RSI was originally derived from tumor cell-intrinsic gene expression in vitro in a panel of cancer cell lines,^10^ analysis of published single-cell RNA-sequencing data from human breast cancer^31^ shows expression of RSI genes among malignant and diverse nonmalignant cells within the tumor microenvironment (Fig. 1F). The RSI genes *IRF1, STAT1*, and *PRKCB*, which were strongly associated with sTIL infiltration, showed enriched (*IRF1* and *STAT1*) or nearly exclusive (*PRKCB*) expression in lymphoid and myeloid immune cells (Fig. 1G). The single-cell expression pattern of these genes in the breast and our finding that their co-expression in pretreatment TNBC samples is highly correlated with sTIL infiltration suggests that RSI scores are strongly influenced by the degree of immune infiltration within individual tumors.

### RSI correlates with specific patterns of leukocyte infiltration in TNBC

**Fig. 2.**
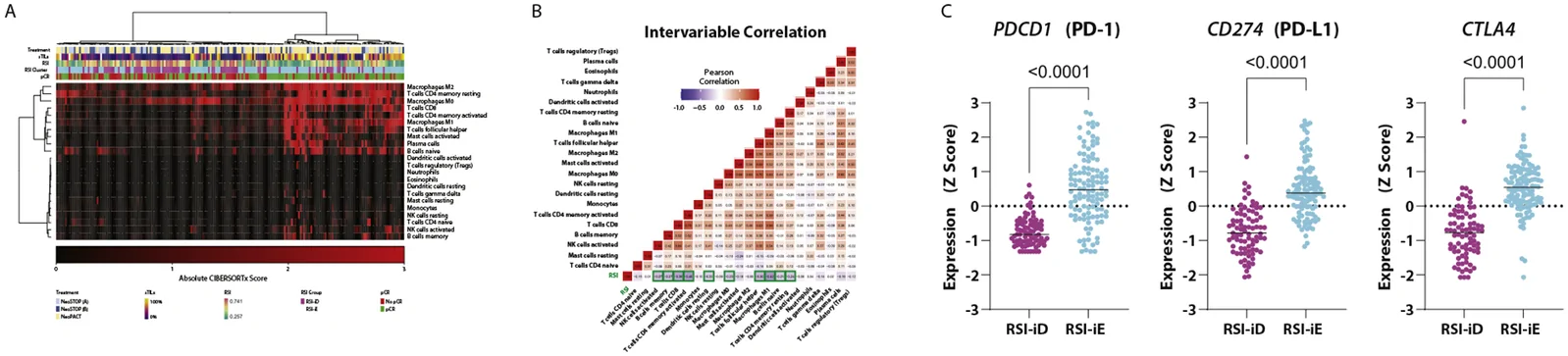
(A) Unsupervised hierarchical clustering of 197 pretreatment TNBC samples by absolute CIBERSORTx leukocyte fractions. (B) Pearson correlation coefficients among CIBERSORTx leukocyte fractions, and RSI. Cells with green outline denote significant (BH *q* < 0.01) associations with RSI. (C) Expression of *PDCD1* (PD-1), *CD274* (PD-L1), and *CTLA4* in pre-treatment TNBC samples by RSI-iD and RSI-iE clusters; comparison is BH corrected *q* value. *Abbreviations:* pCR = pathologic complete response; RSI = radiosensitivity index; RSI-iD = RSI immune-depleted; RSI-iE = RSI immune-enriched; sTIL = stromal tumor-infiltrating lymphocyte; TNBC = triple-negative breast cancer.

To understand how RSI correlates with discrete leukocyte populations within the TNBC microenvironment, we performed CIBERSORTx leukocyte deconvolution^29^ on RNA-sequencing data. Using absolute fraction analysis, the RSI-iE cluster showed enrichment of a leukocyte cluster characterized by increased abundance of several populations (Fig. 2A). Correlation analysis showed significant inverse associations between RSI and M0 and M1 macrophages, CD4+ memory resting, CD4+ memory activated, CD8+, and follicular helper T-cells, activated natural killer cells, naïve and memory B cells, and resting dendritic cells (Fig. 2B). Because of the significant enrichment in T-cell populations in the RSI-iE cluster, we also sought to determine if there was evidence of T-cell exhaustion and found that expression of the immune checkpoint blockade molecules *PDCD1* (PD-1), *CD274* (PD-L1), and *CTLA4* were all significantly elevated in the RSI-iE compared with the RSI-iD cluster (Fig. 2C).

### Posttreatment changes in RSI and predicted radiosensitivity

**Fig. 3.**
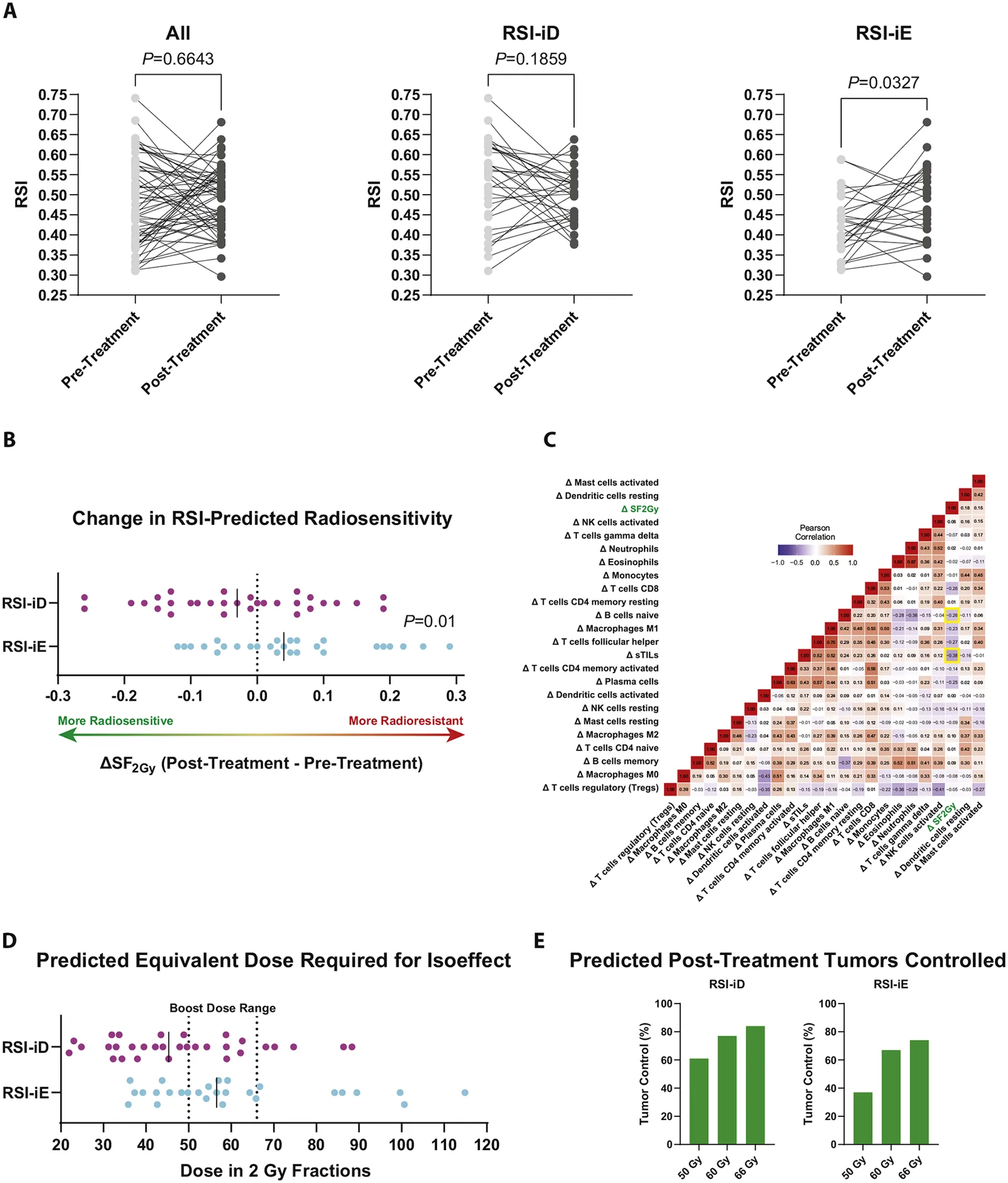
(A) Comparison of paired pretreatment and posttreatment RSI within all samples and the RSI-iD and RSI-iE clusters; *P* is paired *t* test. (B) Plot of change in RSI predicted SF_2Gy_ between posttreatment and paired pretreatment samples by RSI-iD and RSI-iE cluster. *P* is *t* test. (C) Pearson correlation coefficients among change in CIBERSORTx leukocyte fractions, change in sTILs, and change in SF_2Gy_. Cells with yellow outline denote trend (*P* < .05, but not *q* < 0.01) associations with change in SF_2Gy_. (D) Plot of equivalent dose required to achieve tumor control isoeffect in paired posttreatment compared with pretreatment samples by RSI-iD and RSI-iE cluster. (E) Predicted fraction of posttreatment tumors controlled with 50, 60, and 66 Gy at 2 Gy per fraction in RSI-iD and RSI-iE clusters. *Abbreviations:* RSI = radiosensitivity index; RSI-iD = RSI immune-depleted; RSI-iE = RSI immune-enriched; sTIL = stromal tumor-infiltrating lymphocyte.

Paired pre- and posttreatment (residual disease) RNA-sequencing data were available for 58 of 84 (69%) patients who did not achieve a pCR. Paired posttreatment sequencing data were unavailable for 26 patients because of unavailability of the surgical specimen (n = 4), residual disease that deemed on internal pathology review (n = 13) or external pathology review (n = 8) to be too small or of insufficient cellularity to yield tissue for RNA sequencing, and not proceeding to surgery because of disease progression while on neoadjuvant systemic therapy (n = 1). When comparing paired pre- and posttreatment RSI, there was no significant difference in the entire cohort ($\bar{\Delta }=0.007$; *P* = .66) or in patients whose pretreatment tumors were in the RSI-iD cluster ($\bar{\Delta }=-0.030$; *P* = .19), but in patients whose pretreatment tumors were in the RSI-iE cluster, there was a significant increase in RSI ($\bar{\Delta }=0.049$; *P* = .03) in posttreatment compared with pretreatment samples (Fig. 3A). We calculated the predicted difference in tumor cell survival after an acute exposure of 2 Gy (ΔSF_2Gy_) in patients whose pretreatment tumors were in the RSI-iD and RSI-iE clusters. There was a significant difference in change in predicted radiosensitivity between posttreatment and pretreatment RSI-iD and RSI-iE tumors, with posttreatment RSI-iD tumors trending toward greater radiosensitivity and posttreatment RSI-iE tumors trending toward greater radioresistance compared with their paired pretreatment tumors (*P* = .01) (Fig. 3B). For robustness, a linear mixed model was used to test if systemic therapy and RSI cluster identity significantly predicted change in RSI and ΔSF_2Gy_, accounting for the 26 patients without pCR for whom we did not have a paired residual disease sample for analysis. We found that RSI cluster identity significantly predicted change in RSI and ΔSF_2Gy_ from pretreatment to posttreatment (F (1,138) = 22.35; *P* < .001)) but neoadjuvant systemic therapy did not (F(1,138) = 1.43; *P* = .23)). In total, 29 of the 58 patients (50%) with matched tumor specimens exhibited higher predicted radioresistance in the posttreatment compared with pretreatment sample. To determine if a change in SF_2Gy_ was associated with any therapy-related changes in the tumor immune microenvironment, we calculated the absolute change in CIBERSORTx leukocyte fractions and sTILs in posttreatment compared with pretreatment tumors. This analysis showed relatively coordinated changes in several T-cell fractions (CD8+, CD4+ memory activated and resting, and follicular helper), B-cell fractions (naïve and plasma cells), and M1 macrophages, and positive associations between all of these and a change in sTILs (Fig. 3C). There was an inverse association between change in sTILs and ΔSF_2Gy_, and between change in naïve B cells and ΔSF_2Gy_, although these associations were not significant after adjusting for multiple comparisons. We suggest that loss of these populations in the posttreatment tumor may lead to increased radioresistance. To illustrate the change in radiation dose that would be required to achieve equivalent control between pretreatment and posttreatment samples, the maximum number of initial clonogens (*N*) that could be inactivated by 25 fractions of 2 Gy in each pretreatment tumor and achieve 5-year probability of control (*P*) of 94.7%^34^ was calculated using each pretreatment tumor’s predicted SF_2Gy_ according to $N=\frac{-\text{ln}(0.947)}{{{\text{SF}}_{2\text{Gy},\text{Pre}}}^{25}}$. The corresponding dose (*D*) in 2-Gy fractions to achieve the same probability of control for that tumor’s number of clonogens using the paired posttreatment predicted SF_2Gy_ was then calculated according to $D=2\left(\frac{\text{ln}\left(-\frac{\text{ln}(0.947)}{N}\right)}{\text{ln}\left({\text{SF}}_{2\text{Gy},\text{Post}}\right)}\right)$ and is shown in Figure 3D. Compared with 50 Gy in 2-Gy fractions to the pretreatment tumor, doses of 50, 60, and 66 Gy to the paired posttreatment tumor would result in equivalent control for 61%, 77%, and 84% of RSI-iD tumors, respectively, and 37%, 67%, and 74% of RSI-iE tumors, respectively (Fig. 3E).

## Discussion

In this manuscript, we have assessed the association between RSI, a validated genomic predictor of radiosensitivity, and the immune microenvironment in paired pretreatment and posttreatment residual disease TNBCs. In pretreatment specimens, we observed that unsupervised clustering of the 10 RSI genes clearly identifies RSI-iE and RSI-iD subsets that have markedly different levels of stromal leukocyte infiltration, differential expression of immune checkpoint molecules, and significantly different rates of response to neoadjuvant systemic therapy. It is well established that baseline immune enrichment, whether defined by sTILs or a variety of gene expression signatures, is associated with improved responses to various antineoplastic therapies.^28,33^ The interaction between immune enrichment and radiation therapy, and the dependence of radiation therapy on the immune response is less well understood.^35^ Previous work has reported that RSI is associated with distinct immune microenvironment characteristics that predict response to radiation therapy across a spectrum of solid malignancies.^36^ Our analysis shows that immune enrichment, as identified by RSI, similarly predicts response to neoadjuvant systemic therapy in TNBC. Our analysis also highlights that the immune composition of the tumor is expected to strongly influence calculation of the RSI. This is an important finding, since RSI was derived from tumor cell-intrinsic gene expression in vitro in a panel of cancer cell lines,^10^ without considering the heterogeneous composition of the tumor microenvironment.

**Fig. 4.**
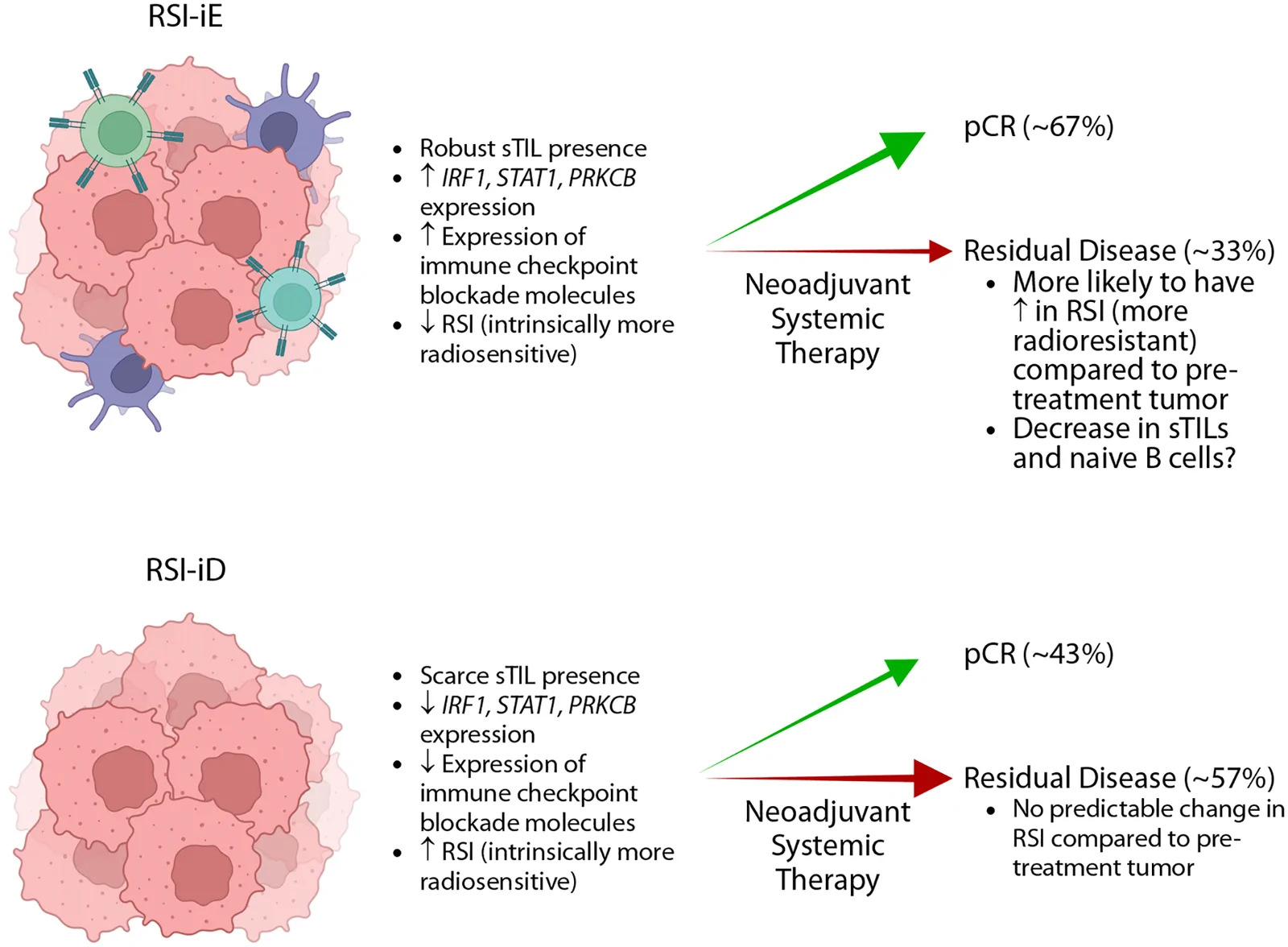
Baseline characteristics of RSI-iE and RSI-iD TNBC, and impact of neoadjuvant systemic therapy on RSI and predicted radiosensitivity in patients with RSI-iE and RSI-iD with residual disease. *Abbreviations:* pCR = pathologic complete response; RSI = radiosensitivity index; RSI-iD = RSI immune-depleted; RSI-iE = RSI immune-enriched; sTIL = stromal tumor-infiltrating lymphocyte; TNBC = triple-negative breast cancer.

The availability of paired posttreatment residual disease samples allowed us to investigate how RSI changes during neoadjuvant systemic therapy. On paired sample analysis, we found that RSI did not have a predictable pattern of change across the entire cohort with paired samples or among patients with RSI-iD. However, among patients with RSI-iE, there was a significant increase in RSI in paired posttreatment compared with pretreatment tumors. Although this failed to maintain significance after adjusting for multiple comparisons, we also found that therapy-induced reduction of histologically quantified sTILs was associated with higher predicted radioresistance, and CIBERSORTx suggests that loss of naïve B cells may be an important driver of this phenomenon. Using a model in which we normalize clonogenic sterilization rates between pretreatment tumors, we show that the RSI predicts that a significant fraction of paired posttreatment tumors would fail to achieve an equivalent level of control if treated with the same dose or even a supplemental boost dose. Interestingly, this effect was even more pronounced for RSI-iE tumors with residual disease, suggesting that the presence of residual disease in the context of an initially immune-enriched tumor is an indicator of higher intrinsic radioresistance (Fig. 4). This is congruent with a recent analysis showing that high baseline sTILs do not confer an event-free survival benefit in patients who do not achieve pCR.^37^ Since most patients with TNBC receive systemic therapy in the neoadjuvant setting, this has important implications for potential clinical use. These findings should be validated and confirmed in other trial data sets with availability of paired pre- and posttreatment tissue samples.

Completed and ongoing randomized trials have focused on eliminating radiation therapy in patients with breast cancer based on clinicopathologic features alone or in combination with response to neoadjuvant therapy (eg, NRG-BR008 [NCT05705401] and NSABP B-51/RTOG 1304) or clinicopathologic features in combination with genomic classifiers (eg, NRG-BR007 [NCT04852887] and CCTG MA.39 [NCT03488693]). The published findings from NSABP B-51/RTOG 1304 confirm that pCR to neoadjuvant systemic therapy is a reliable indicator of locoregional recurrence risk and benefit from adjuvant radiation therapy.^9^ Conversely, it is known that the presence of residual disease after neoadjuvant systemic therapy is associated with significantly increased risks of both locoregional and distant metastatic recurrence.^26^ Although radiation therapy doses after neoadjuvant systemic therapy are often prescribed based on pretreatment clinical distribution of disease, radiographic and/or pathologic evidence of treatment response, and extent of surgery, our results suggest that integration of therapy-induced genomic and immunologic changes may allow a more personalized approach to radiation therapy.

## Figures and Tables

**Table 1:** Clinical, tumor, and treatment characteristics

Characteristic	Total (N = 197)	NeoSTOP (N = 88)	NeoPACT (N = 109)	*P*
Age, median (range)	51 (27–70)	51 (31–70)	51 (27–70)	.49
Race				.19
White	150 (76.1%)	65 (73.9%)	85 (78.0%)	
Black	37 (18.8%)	17 (19.3%)	20 (18.3%)	
Asian	5 (2.5%)	2 (2.3%)	3 (2.8%)	
Other	4 (2.0%)	4 (4.5%)	0 (0.0%)	
Unknown	1 (0.5%)	0 (0.0%)	1 (0.9%)	
Ethnicity				1.00
Non-Hispanic/Latino	192 (97.5%)	86 (97.7%)	106 (97.2%)	
Hispanic/Latino	5 (2.5%)	2 (2.3%)	3 (2.8%)	
T stage category				.10
T1	38 (19.3%)	18 (20.5%)	20 (18.3%)	
T2	131 (66.5%)	62 (70.5%)	69 (63.3%)	
T3	27 (13.7%)	7 (8.0%)	20 (18.3%)	
T4	1 (0.5%)	1 (1.1%)	0 (0.0%)	
Nodal status				.45
Negative	128 (65.0%)	60 (68.2%)	68 (62.4%)	
Positive	69 (35.0%)	28 (31.8%)	41 (37.6%)	
sTILs %, median, range*	15 (1–95)	15 (1–95)	17.5 (1–95)	1.00
RSI cluster				.56
RSI-iD	81 (41.1%)	34 (38.6%)	47 (43.1%)	
RSI-iE	116 (58.9%)	54 (61.4%)	62 (56.9%)	
Systemic therapy^†^				<.001
CbP + AC	43 (21.8%)	43 (48.9%)	0 (0.0%)	
CbD	45 (22.8%)	45 (51.1%)	0 (0.0%)	
CbD + Pembrolizumab	109 (55.3%)	0 (0.0%)	109 (100.0%)	
Pathologic complete response				1.00
No	84 (42.6%)	38 (43.2%)	46 (42.2%)	
Yes	113 (57.4%)	50 (56.8%)	63 (57.8%)	

*Abbreviations:* RSI = radiosensitivity index; RSI-iD = RSI immune-depleted; RSI-iE = RSI immune-enriched; sTIL = stromal tumor-infiltrating lymphocyte.

* Missing for 5 patients (NeoSTOP n = 4; NeoPACT n = 1).

† CbP + AC = carboplatin (AUC6) every 3 weeks × 4 plus paclitaxel (80 mg/m^2^) weekly × 12 followed by doxorubicin (60 mg/m^2^) plus cyclophosphamide (600 mg/m^2^) every 2 weeks × 4; CbD = carboplatin (AUC6) plus docetaxel (75 mg/m^2^) every 3 weeks × 6; CbD + pembrolizumab = CbD (as before) plus pembrolizumab (200 mg) every 3 weeks × 6.

## Data Availability

Any requests for anonymized trial data or supporting material will be reviewed on a case-by-case basis. Only requests that have scientifically and methodologically sound proposals will be considered, and the usage of the shared trial data or supporting material will be limited to the approved proposal. The final decision as to whether data or supporting material might be shared and the exact data or supporting material to be shared will be made between the trial team and the principal investigators. Proposals should be directed to the corresponding author.
